# Correction

**DOI:** 10.1080/2162402X.2026.2627753

**Published:** 2026-02-13

**Article Title:** Human NK cells activated by EBV+ lymphoblastoid cells overcome anti-apoptotic mechanisms of drug resistance in haematological cancer cells

**Author:** Sánchez-Martínez, D., Azaceta, G., Muntasell, A., Aguiló, N., Núñez, D., Gálvez, E.M., Naval, J., Anel, A., Palomera, L., Vilches, C., Marzo, I., Villalba, M., and Pardo, J.

**Journal**: *OncoImmunology*

**Bibliometrics**: Volume 4, Issue 3, Article e991613.

**DOI:** 10.4161/2162402X.2014.991613

In the original published article, the same image was inadvertently used in Figure 2D to represent the activated NKp44 dot plot and the NKp46 dot plot. When concerns were raised regarding the similarities, the authors reviewed their data and noticed the error, working with the Publisher to rectify the issue and provide the correct image for the NKp44 dot plot. This error has no impact on the interpretation of the results, nor does it influence the conclusions of the work.

The correct image is reproduced below for transparency.

**Figure:**
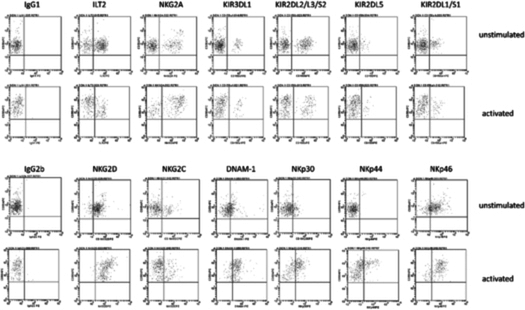

The authors apologize for any inconvenience that may have been caused.

